# Current Status of Mycotoxin Contamination of Food Commodities in Zimbabwe

**DOI:** 10.3390/toxins10050089

**Published:** 2018-05-03

**Authors:** Nancy Nleya, Modupeade Christianah Adetunji, Mulunda Mwanza

**Affiliations:** 1 Department of Animal Health, Northwest University, Mafikeng, Private Bag X2046, Mmabatho 2735, South Africa; ogunrinumodupe@gmail.com (M.C.A.); Mulunda.Mwanza@nwu.ac.za (M.M.); 2 Department of Applied Biology and Biochemistry, National University of Science and Technology, P.O. Box AC 939 Ascot, Bulawayo, Zimbabwe; 3 Department of Biological Sciences, McPherson University, Seriki Sotayo, Ogun State, Abeokuta P.M.B. 2094, Ogun State, Nigeria

**Keywords:** mycotoxins, legislation, Zimbabwe, food, feed, standards, analysis

## Abstract

Agricultural products, especially cereal grains, serve as staple foods in sub-Saharan Africa. However, climatic conditions in this region can lead to contamination of these commodities by moulds, with subsequent production of mycotoxins posing health risks to both humans and animals. There is limited documentation on the occurrence of mycotoxins in sub-Saharan African countries, leading to the exposure of their populations to a wide variety of mycotoxins through consumption of contaminated foods. This review aims at highlighting the current status of mycotoxin contamination of food products in Zimbabwe and recommended strategies of reducing this problem. Zimbabwe is one of the African countries with very little information with regards to mycotoxin contamination of its food commodities, both on the market and at household levels. Even though evidence of multitoxin occurrence in some food commodities such as maize and other staple foods exist, available published research focuses only on *Aspergillus* and *Fusarium* mycotoxins, namely aflatoxins, deoxynivalenol (DON), trichothecenes, fumonisins, and zearalenone (ZEA). Occurrence of mycotoxins in the food chain has been mainly associated with poor agricultural practices. Analysis of mycotoxins has been done mainly using chromatographic and immunological methods. Zimbabwe has adopted European standards, but the legislation is quite flexible, with testing for mycotoxin contamination in food commodities being done voluntarily or upon request. Therefore, the country needs to tighten its legislation as well as adopt stricter standards that will improve the food safety and security of the masses.

## 1. Introduction

Mycotoxins are secondary metabolites produced mainly by moulds belonging to the genera *Aspergillus*, *Fusarium*, and *Penicillium* [1] that contaminate food commodities worldwide, with developing countries being most affected [2]. Different fungal species produce different mycotoxins [3] which are named on the basis of the mould that produces them; for example, aflatoxins are produced by *Aspergillus*, whereas fumonisins are produced by *Fusarium*. More than 300 mycotoxins have been identified [4], but those of significant toxicological importance are aflatoxins, zearalenone, trichothecenes, fumonisins, and ochratoxins [5,6,7,8], as they possess carcinogenic and immunosuppressive properties and can also retard growth in both humans and animals, thereby affecting productivity [9].

Mycotoxins are very stable, and their contamination in agricultural commodities can be pre-harvest, during harvest, and post-harvest [7,10,11], resulting in the classification of the mycotoxin-producing fungi as field and storage fungi. Mycotoxin production is affected by several conditions which can be biological, chemical, and physiological [12]. However, some of these factors are beyond human control, especially ecological and environmental factors such as temperature, nutrient content of substrate, and relative humidity, which play important roles in toxin production. Most mycotoxins are produced in hot and humid climates, mainly the tropical countries [4], most of which are African countries [6].

Humans are exposed to mycotoxins through consumption of foods of plant origin that may be contaminated with toxins or as mycotoxin metabolites which are carried over into animal food products [4]. Ingestion of mycotoxins results in a disease known as mycotoxicosis in animals and humans [4,11,13]. The toxicity of mycotoxins on specific tissues and organs depends on the toxin involved, concentration of the toxin, and the age and nutritional status of the individual [3,13]. Some mycotoxins affect the nervous system, some cause liver and kidney damage, and others even cause vomiting in some animal species, with clinical implications ranging from chronic disease to death.

Biodiversity of mycotoxin-producing fungi in the environment results in a wide range of mycotoxins being present in some agricultural commodities, known as multitoxin occurrence [14], with moulds being capable of producing multiple mycotoxins [4]. Multitoxin contamination of food commodities results in more severe effects when compared to single toxin contamination due to synergism [15]. This review focused on research that has been done in Zimbabwe on mycotoxins from their discovery to date.

## 2. Methods

The review was done under Preferred Reporting Items for Systematic Reviews and Meta-Analyses (PRISMA) guidelines [16] in order to gather information on mycotoxin contamination of foods and feeds in Zimbabwe. Key words/phrases used to access information were: mycotoxin, aflatoxin, food, stock feed, feed ration, cereal grains, oil seeds, legumes, legislation, analysis, tolerance limits, maize, sorghum, mealie-meal, groundnuts, silage, milk, livestock, poultry, cattle, *Aspergillus*, Zimbabwe, and sub-Saharan Africa. Papers with relevant information were downloaded and analysed. A total of 81 papers were identified and 31 of these had information relevant to the review.

## 3. Occurrence of Toxigenic Fungi in Foods and Feeds in Zimbabwe

Little information is available on the typical range of mycotoxins in foods that are mainly consumed in Zimbabwe [1,17]. However, considerable work on mycotoxins has been done in Zimbabwe. Research has revealed the presence of mycotoxin-producing moulds on maize, the staple food of Zimbabwe which constitutes about 70% of the diet, and other cereal crops such as wheat, finger millet, millet, and sorghum [6,17,18,19,20,21,22,23]. The most common genera that were isolated are *Aspergillus* and *Fusarium* [22,23], which are known to produce mycotoxins of concern to animal and human health, namely aflatoxins, zearalenone, trichothecenes, fumonisins, and ochratoxins [24]. Colonisation of the grains by the moulds is mainly due to infection of the cobs in the field by insects such as the maize stalk borer (*Busseola fusca*), inadequate drying of crops, as well as the primitive methods used during the shelling of maize and other grains [1,19,25].

Other researchers have worked on groundnuts, beans, cowpeas, and Bambara nuts, where they also isolated *Aspergillus* and *Fusarium* [21,26,27]; whereas in another study [2], *Aspergillus flavus* (*A. flavus*) and *Aspergillus parasiticus* (*A. parasiticus*) were isolated from peanut butter samples from Zimbabwe and Botswana. This suggests that the temperature of 160 °C used for roasting in commercial peanut butter production [28] is not high enough to destroy the vegetative cells and spores, not to mention the temperatures used in homemade peanut butter, which are not even monitored [2]. This increases the chances of continual aflatoxin production in the peanut butter during storage.

A 120-day storage trial carried out by Titterton [29] to assess the effects of different storage conditions on the nutritional value of maize and sorghum stovers showed a positive effect of storage on the nutritional values. In addition, several potential mycotoxin-producing moulds belonging to the genera *Fusarium*, *Phoma*, *Altenaria*, *Penicilium*, and *Aspergillus* were also isolated from the stovers [30]. *Aspergillus* and *Penicillium* species were detected in both freshly harvested and stored samples. During the dry season, farmers face the challenge of feed shortage [29], hence the use of supplements such as plant stovers. The presence of mycotoxigenic fungi in the stovers is of concern as this may expose the livestock to mycotoxin poisoning and can also be carried over into foods of animal origin, followed by subsequent transfer along the food chain to humans, causing harm [31].

## 4. Aflatoxins

A lot of attention has been given to aflatoxins due to their effect on animal and human health [7], which ranges from gastroenteritis to cancers in acute and chronic aflatoxicosis, respectively. Aflatoxins are produced by moulds belonging to the genus *Aspergillus*, dominated by *A flavus*, *A parasiticus*, and *A nomius*. There are four naturally occurring aflatoxins in food and feed, namely aflatoxin (AF) B_1_, B_2_, G_1_, and G_2_, depending on the fluorescence colour under ultraviolet light at 365 nm [32,33,34]. AFB_1_ is the most toxic of them all and is produced by almost all aflatoxigenic *Aspergillus* species, whereas there are some species that cannot produce the G series, such as *A flavus* (produces B_1_ and B_2_ only) [35,36,37,38]. When consumed, aflatoxins cause liver cancer and stunted growth as well as suppressing immunity. In the body, aflatoxins are biotransformed predominantly in the liver, and the by-products or metabolites are eventually excreted mainly through renal flux [39]. When lactating cows and other mammals ingest aflatoxins in contaminated feed, toxic metabolites can be formed and found in milk due to the biotransformation of AFB_1_ and AFB_2_ by the hepatic microsomal mixed-function oxidase system to a hydroxylated metabolite known as “milk toxin” or aflatoxin M_1_ (AFM_1_) and AFM_2_, respectively, which is excreted in the milk and urine [40]. It has been shown that AFM_1_ retains some carcinogenicity, thus exposing humans to chronic toxicity, and thus making milk a source of aflatoxin contamination in humans [41].

## 5. Aflatoxins in Food and Feed

Research on aflatoxins in Zimbabwe was initiated by disease outbreaks on farms, particularly in poultry. One such particular incident was the death of 70 ostriches on a farm. Analysis of the feed showed contamination by aflatoxin of 11 µg/kg. Samples from other feed batches were also tested and gave results as high as 129 µg/kg. However, no action was taken as there are no limits for ostrich feed [42]. The presence of determinants of aflatoxin exposure in the Zimbabwean population has been used as indicators of the people’s exposure to aflatoxin poisoning [43]. This has been revealed by the presence of AFM_1_ in urine and breast milk of the rural populations [43,44,45].

Analysis of some food commodities and their products showed the presence of aflatoxins [2,27,28]. Foods that have been analysed include maize, groundnuts, peanut butter, beans, Bambara nuts, and cowpeas, among others, as shown in Table 1. Probst et al. [46] analysed maize samples from African countries including Zimbabwe, and all the samples tested positive for aflatoxin contamination, ranging from 9–123 µg/kg, which is above the Codex Alimentarius Commission permissible limit of 5 µg/kg for maize intended for human consumption [47]. Siwela [48] also analysed maize and maize meal samples intended for human consumption, and 35% of the samples were above the Zimbabwean 20 µg/kg limit, with one sample having a concentration of 1391 µg/kg.

The presence of both *Aspergillus* and aflatoxins in peanut butter is of great concern as it is considered a highly nutritious food recommended for infants [53]. Infants are more susceptible to the effects of mycotoxins [54]. Aflatoxin presence in the peanut butter could be attributed to their high melting points, which range from 237 °C to 289 °C [55]. The temperatures used during peanut roasting are not high enough to destroy them, hence their presence in the final product. This was shown by the presence of aflatoxins in peanut butter at levels that were above the 4 µg/kg European Union (EU) limit and some above the Zimbabwean legislation of 15 µg/kg for total aflatoxin [2,48,56].

All four major aflatoxins were detected in groundnuts, Bambara nuts, beans, and cowpeas in research done by Maringe and others [27]. Groundnuts and other legumes are important protein supplements in animal feeds [57,58,59] as well as the human population [48]. Traces of aflatoxins may remain in the seedcake, which is usually used as feed [42]. Therefore, it is of paramount importance to assess the levels of aflatoxin contamination of these seeds so as to avoid or reduce carryover into foods of animal origin such as milk, eggs, and meat, among others.

There is only one published study with regards to aflatoxin contamination of milk (AFM_1_) in Zimbabwe, carried out by Choga et al. [50]. Milk and dairy products, as important constituents in the human diet, may be the major way for entrance of aflatoxins into humans if not monitored [60]. Crop residues have been used as alternative animal feeds [61], and research has indicated the presence of aflatoxigenic moulds with potential to produce mycotoxins which can be carried over into foods of animal origin such as milk. Findings by Morton and coworkers [52] on the effects of post-harvest practices on the production and nutritive value of maize residues in Zimbabwe indicated the presence of AFB_1_ in the maize stovers used to feed dairy cows in the Silozwi Communal Area area south of Bulawayo, although the concentrations were low. Milk is consumed mainly by infants, especially during the weaning period, thus the growth of infants and young children could be impaired by the presence of AFM_1_ in milk [43]. Nevertheless, there are no reports on mycotoxin contamination of milk products.

## 6. *Fusarium* Mycotoxins

The major mycotoxins produced by *Fusarium* species are trichothecenes, zearalenone (ZEA), and fumonisins [8,62], and can cause several acute and chronic mycotoxicosis in humans [63]. *Fusarium* species are classified under field fungi as they tend to contaminate food crops before harvest [64,65].

Fumonisins are toxic compounds produced predominantly by *F. verticillioides*, previously known as *F moniliforme* and *F. proliferatum*, and other toxigenic *Fusarium* species [17,21,64]. They have been associated with oesophageal cancer in South Africa [66]. Fumonisin B_1_ (FB_1_), the most toxic of the fumonisins, has been shown to interfere with folate uptake, resulting in congenital disorders in human babies such as neural tube defects [4].

Work done on *Fusarium* mycotoxins on Zimbabwean foods is shown in Table 2. Other researchers [17] reported on fumonisin contamination on all the 388 samples of maize grain collected from households in Shamva and Makoni rural areas. Even though the concentrations were below the 1000 µg/kg European Community (EC) limit, there is still the risk of the development of chronic ailments such as cancer in these populations. Research by Mubatanhema and others [19] revealed coproduction of zearalenone, moniliformin, and fumonisin B_1_ by *Fusarium* isolates from maize samples collected from nine grain marketing board (GMB) centres in Zimbabwe. There has been limited research in Zimbabwe on other *Fusarium* toxins such as trichothecenes and zearalenone, which has also been the case with other countries [8]. Multitoxin production by mycotoxigenic fungi in Zimbabwean food samples was also demonstrated by Gehesquière and collegues [20] and Murashiki and coworkers [17]. Fumonisins were also shown to be the prevalent mycotoxin in the maize stovers used as feed during the dry season [52].

## 7. Mycotoxin Analysis

Mycotoxin poisoning can occur even after consumption of very small quantities of the toxin, therefore there is a need to use sensitive methods for their analysis and quantification [69,70]. The most common methods for mycotoxin analysis are chromatographic, such as thin-layer chromatography (TLC), high-performance liquid chromatography (HPLC), and immunoassays (radioimmunoassays, RDIs, and enzyme immunoassays, EIAs) [69]. Chromatographic methods combined with mass spectrometry have also been recently used in the detection and quantification of mycotoxins [71], and are capable of multitoxin detection [69,72] and even that of masked mycotoxins. Analysis of food and feed matrices from Zimbabwe has been carried out using some of these methods as shown in Table 3.

## 8. Legislation

Many countries are recognising the importance of aflatoxins and have generated regulations governing permissible concentrations of aflatoxins in food depending on the intended use of the particular food involved. These limits are, however, guided by the food regulating bodies such as the Food and Agriculture Organization (FAO), World Health Organization (WHO), and EU. The limits for individual countries range from 4–20 µg/kg [7] for foods intended for human consumption; whereas for milk and milk products, the range is 0.05–1.0 µg/kg. The strictest standards are those for the EU, which are 2 µg/kg for AFB_1_ for human consumption and 5 µg/kg for lactating cows’ feed, 4 µg/kg for total aflatoxin, and 0.05 µg/kg for AFM_1_. There are no regulatory limits set for mycotoxin level in food and feed in Zimbabwe, except for aflatoxins [74], which are not even considered when farmers come to deliver their produce as well as when food producers buy the raw materials [64]. This is the same for most developing countries in Southern Africa, mainly due to the shortage of resources for mycotoxin testing making it difficult to enforce laws on mycotoxin regulation [7]. Zimbabwe initially adopted the 5–25 µg/kg limit for AFB_1_ in food intended for human consumption in 1971; however, it was revised in 1990 to 20 µg/kg [42]. Politics and economic crisis have made it tough to come up with an up-to-date and effective food control system [75]. When the GMB was responsible for handling agricultural commodities, it was easy to control the movement of produces. No grain would be released before being tested for aflatoxins, either for export or local consumption. However, with the liberalisation and indigenisation of the economy, farmers and manufacturers trade without government interference [42]. This has made the country the only one in the region with a twofold system of food standards, including the voluntary one and the controlled one [76]; one where the processor makes a choice on whether they want the raw commodity tested for contaminants or not, and one where the analysis of produce is mandatory. Zimbabwe has been affected by droughts which caused food shortages, and coupled with economic hardships, its citizens now worry about having food on the table, without being concerned too much about quality. In the case of livestock poisoning, there are no rules regarding aflatoxin limits in feed, and this has seen these cases settled out of court between the feed manufacturer and the farmer [42].

## 9. Discussion

This review has shown that the Zimbabwean population and livestock are exposed to a wide range of mycotoxins, as evidenced by multitoxin occurrence in some of the foods and feeds [19,20,52], which exposes the populations to more severe toxicity and immune suppression as compared to single mycotoxin contamination [63]. Work done by Siwela [48] showed that the aflatoxin contamination in 69% of the samples were within the limit of 20 µg/kg, with the remaining 31% above the limit. These results concur with those that were reported in Kenya, where 96% of the groundnut samples were contaminated with <4 µg/kg of AFB_1_, while 4% were above the national regulatory limit of 20 µg/kg [77]. Although most of the samples are within the limits, the population is still at risk of chronic aflatoxicosis, which could lead to cancer development later in life and immunosuppression, as well as stunted growth in children and animals [78]. However, a study in the Democratic Republic of Congo showed that a greater percentage of the groundnut samples analysed (70%) had aflatoxin contamination of ˃5 µg/kg [79]. Foods such as groundnuts and legumes are used as alternative protein sources [53] and their contamination by aflatoxins is of great concern, especially to children and vegetarians. Research on maize showed that the most common mycotoxins are aflatoxins and fumonisin B_1_ [67], as the maize crop is known to be susceptible to fumonisin B-producing moulds. The EU maximum limit for fumonisins in food intended for human consumption is 1000 µg/kg [80], and most of the samples that were analysed had figures above this limit (Table 2). Gehesquière and others [20] showed that the levels of mycotoxins increase mainly during storage compared to pre-harvest. This was supported by Mubatanhema and coworkers [19], who attributed mould infection of maize as being a result of primitive methods used when shelling the grain which damage the kernels, paving the way for fungal infection. Therefore, there is need for the use of storage facilities that result in minimal growth of mycotoxigenic species. However, for groundnuts, contamination was mainly before harvest, with no significant change during storage [28]. Very little relevant research has been done on milk, even though it is an important component of the diet, especially used for weaning of children. The limited information on contamination levels of AFM_1_ in milk and milk products means that the country is still very far from dealing with stunted growth in children, as mycotoxins have been associated with growth suppression [43,54,81].

The bulk of the research on mycotoxins in Zimbabwe was conducted between 1980 and the early 1990s, and most of the samples analysed were taken from the GMB. This might not have given a true reflection on the extent of food contamination, as farmers have a tendency of sending the best produce to the GMB so that they can earn more money, whereas what would remain for their consumption will be of compromised quality. The majority of research done after 2000 has been conducted outside the country, due to limited funds and equipment making it difficult for the authorities involved in policy formulation to access this information. Also, most journals with high impact factors would not publish research done in most developing countries, as they lag behind in technological advancement in terms of analytical methods. In addition, most researchers from developing countries cannot afford the exorbitant publication fees charged by most journals. This means that most of the research findings remain unpublished or get published after several years.

## 10. Conclusions

Most of the work done in Zimbabwe has been focused on *Aspergillus* and *Fusarium* mycotoxins and has shown the presence of these mycotoxins in foods and feeds. Therefore, there is a need to come up with strategies that will help reduce and manage levels of the mycotoxins in foods. Zimbabwe does not have legislation on the maximum levels of mycotoxins, but adopts European legislation [20]. This is a cause of concern, especially with regards to maize as it is used as an animal feed in Europe, whereas in Zimbabwe it is the staple food. Zimbabwe should come up with its own standards which cater for the nation’s own diet. For legumes, there are no limits on the level of mycotoxins, making it difficult to enforce law [27]. There is also a need for researchers to share their findings with the general population. A study by Loreen and Moses [82] revealed that most of the people of Dora village in Mutare did not know anything about aflatoxins, except for the agricultural extension officers, meaning that these officers are not passing on information to the farmers. Research has also indicated that mycotoxin contamination of smaller grains is lower compared to that of the large grains, with some having no mycotoxins at all [21,22,23], so growing smaller grains could be one of the options in trying to reduce the mycotoxin burden; a replacing grains that are highly susceptible to mycotoxin contamination (such as maize) with grains that are less susceptible (such as sorghum or finger millet) could be another option. This will in turn reduce the rate of exposure of consumers to mycotoxins in their diet, especially for children. Improvement on the methods of harvest as well as sorting of the grains before storage will also help in reducing and managing mycotoxins in the granaries [20]. Dehulling, although not nutritionally recommended [83], has been proven as one of the ways of reducing mycotoxin contamination in grains [73,84].

## Figures and Tables

**Table 1:** Occurrence of aflatoxins in foods and feeds in Zimbabwe.

Food/Feed	Mycotoxin	Sample Source	Concentration Range (µg/kg)	Analytical Method	Reference
Groundnut	Total AF	Food and feed companies	˂1–394	HPLC	[48]
Peanut butter	Total AF	Food and feed companies	˂1–213	HPLC	
Cowpeas	Total AF	Food and feed companies	˂1–20	HPLC	
Maize	Total AF	Food and feed companies	˂1–1391	HPLC	
Stock feed	Total AF	Food and feed companies	˂1–30	HPLC	
Cornstarch	Total AF	Food and feed companies	˂1	HPLC	
Beans	Total AF	Food and feed companies	˂1–30	HPLC	
Groundnuts	AFB_1_	Grain marketing board	˂5–250	TLC	[28]
	AFG_1_	Grain marketing board	˂4–200	TLC	
Groundnuts	Total AF	Retail shops and vendors	6.6–622.1	HPLC	[2]
Peanut butter	Total AF	Retail shops and vendors	6.1–247	HPLC	
Groundnuts	AFB_1_	Makoni District	0.7–108.3	HPLC	[27]
	AFB_1_	Shamva District	3.1–175.9	HPLC	
	AFB_2_	Makoni District	1.3–320	HPLC	
	AFB_2_	Shamva District	1.3–320	HPLC	
	AFG_1_	Makoni District	20.9–271.6	HPLC	
	AFG_1_	Shamva District	20.9–271.6	HPLC	
	AFG_2_	Makoni District	29.1–377.8	HPLC	
	AFG_2_	Shamva District	29.1–377.8	HPLC	
	Total AF	Makoni District	9.2–697.8	HPLC	
	Total AF	Shamva District	9.2–697.8	HPLC	
Beans	Total AF	Shamva District	27.3	HPLC	[27]
	Total AF	Makoni District	70.9	HPLC	
Cowpeas	Total AF	Makoni District	1.4–103.4	HPLC	
	Total AF	Shamva District	2.3	HPLC	
Bambara nuts	Total AF	Makoni District	8.6–53.5	HPLC	
Stink bugs	AFB_1_	Bikita District	0.5–0.59	LC-QtoF-MS	[49]
Milk	AFM_1_	Harare	590–4510 *	HPLC	[50]
Maize	Total AF	Zimbabwe	˂1–123	ELISA	[46]
Maize	AFB_1_	Zimbabwe	˂1–11	LC-MS/MS	[51]
Maize	AFB_1_	Shamva District	0.65–26.65	ELISA	[17]
		Makoni District	0.57–9.22	ELISA	
Maize	AFG_1_	Manicaland and Mashonaland Provinces	nd–23.7	HPLC	[20]
Maize stovers	AFB_1_	Silozwi Communal Area	0.8–1.6	HPLC	[52]

* Milk concentration is given in µg/L. HPLC: high-performance liquid chromatography; AF: aflatoxin; TLC: thin-layer chromatography; nd: not detected

**Table 2:** Occurrence of *Fusarium* mycotoxins in foods and feeds in Zimbabwe.

Food/Feed	Mycotoxin	Sample Source	Concentration Range (µg/kg)	Analytical Method	Reference
Maize	Fumonisins	Zimbabwe	36,000–159,000	ELISA	[46]
	DON		nd–12,000	ELISA	
Maize	FB_1_	Zimbabwe	nd–1106	LC-MS/MS	[67]
	DON		nd–492	LC-MS/MS	
Sorghum	Fumonisins	Zimbabwe	8–187	HPLC	[68]
Maize	FB_1_	Shamva District	108.35–337.14	ELISA	[17]
		Makoni District	15.65–579.6	ELISA	
Maize meal	FB_1_	Shamva District	10.43–432.32	ELISA	
		Makoni District	13.84–606.64	ELISA	
Maize (preharvest)	DON	Mainacland and Mashonaland West Provinces	nd–220.6	HPLC	[20]
	ZEA		nd–44.6	HPLC	
	FB_1_		nd–3865.99	HPLC	
	FB_2_		nd–819.01	HPLC	
	FB_3_		nd–472.20	HPLC	
Maize (at harvest)	DON	Mainacland and Mashonaland West Provinces	nd–184	HPLC	
	ZEA		nd–16.3	HPLC	
	FB_1_		nd–3866	HPLC	
	FB_2_		nd–431	HPLC	
	FB_3_		nd–472	HPLC	
Maize (3 months in storage)	DON	Mainacland and Mashonaland West Provinces	nd–147	HPLC	[20]
	ZEA		nd–30.8	HPLC	
	FB_1_		nd–2842	HPLC	
	FB_2_		nd–819	HPLC	
	FB_3_		nd–275	HPLC	
Maize (6 months in storage)	DON	Mainacland and Mashonaland West Provinces	nd–115	HPLC	
	ZEA		nd–44.6	HPLC	
	FB_1_		nd–2709	HPLC	
	FB_2_		nd–424	HPLC	
	FB_3_		nd–121	HPLC	
Maize stovers (beginning of storage)	FB_1_	Silozwi Communal Area	109–752	HPLC	[52]
	ZEA		nd–73		
Maize stovers (end of storage, i.e., 4 months later)	FB_1_	Silozwi Communal Area	nd–38	HPLC	

ZEA: zearalenone; DON: deoxynivalenol; nd: not detected

**Table 3:** Mycotoxin analysis and quantification methods.

Sample	Mycotoxin	Analytical Method	References
Groundnuts	Total Aflatoxin	TLC	[28]
		HPLC	[27]
Maize	Fumonisins	HPLC	[21,73]
	AFB_1_	HPLC	[20]
	AFB_2_	LC-MS/MS	
	AFG_1_	LC-MS/MS	
	AFG_2_	LC-MS/MS	
	FB_1_	LC-MS/MS	
	FB_2_	LC-MS/MS	
	FB_3_	LC-MS/MS	
	ZEA	LC-MS/MS	
	DON	LC-MS/MS	
	OTA	LC-MS/MS	[17]
	AFB_1_	HPLC	
	FB_1_	ELISA	
Urine	AFM_1_	ELISA	[43]
		TLC, HPLC	[44]
Peanut butter	Total aflatoxin	HPLC	[55]
Beans	Total Aflatoxin	HPLC	[27]
Cowpeas	Total Aflatoxin	HPLC	
Bambara nuts	Total Aflatoxin	HPLC	
Milk	AFM_1_	HPLC	[50]

OTA: Ochratoxin A.
